# The persisting burden of invasive pneumococcal disease in HIV patients: an observational cohort study

**DOI:** 10.1186/1471-2334-11-314

**Published:** 2011-11-11

**Authors:** Reed AC Siemieniuk, Dan B Gregson, M John Gill

**Affiliations:** 1 Southern Alberta HIV Clinic, Calgary, Canada; 2 Michael G. DeGroote School of Medicine, McMaster University, Hamilton, Canada; 3 Faculty of Medicine, University of Calgary, Calgary, Canada; 4 Calgary Laboratory Services, Calgary, Canada

## Abstract

**Background:**

The increasing use of highly active antiretroviral therapy (HAART) and pneumococcal immunization along with shifting community exposures may have altered the burden of *Streptococcus pneumoniae *disease in HIV-infected persons. We describe the burden and risk factors for pneumococcal disease in the modern era of HIV care and evaluate the use of a 23-valent pneumococcal polysaccharide vaccine (PPV-23).

**Methods:**

The incidence of invasive pneumococcal disease (IPD) between January 1^st^, 2000 and January 1^st^, 2010 in a regional HIV population in Southern Alberta, Canada was determined by linking comprehensive laboratory and hospital surveillance data. Clinical and epidemiologic data including risk factors for *S. pneumoniae*, history of pneumococcal immunization, serotypes of infections, and length of any hospitalizations for pneumococcal disease were evaluated with multivariate analysis. CD4 count and viral load at immunization were evaluated with a nested case-control analysis.

**Results:**

In 1946 HIV-patients with 11,099 person-years of follow up, there were 68 distinct episodes of pneumococcal disease occurring in 50 patients. Increased risk was seen if female, age >60, Aboriginal ethnicity, lower education, injection drug use, smoking, nadir CD4 <200/μL, chronic obstructive pulmonary disease, and hepatitis C. Overall, the incidence of IPD was 342/100,000 person-years and was reduced to 187/100,000 within three years of PPV-23 immunization (P < 0.01). Although 78% of patients received PPV-23, 74% of IPD episodes were caused by PPV-23 serotypes. In a case-control analysis, HIV viral load at immunization was significantly predictive of PPV-23 failure, while CD4 count was not. 80% of IPD cases required hospitalization: median length of stay was 7 days (range: 1-71); four patients died.

**Conclusions:**

Despite universal access to intensive measures to prevent pneumococcal disease including the widespread use of HAART and PPV-23 immunization, the incidence of IPD remains high in HIV patients with its associated morbidity and mortality.

## Background

*Streptococcus pneumoniae *causes significant morbidity and mortality in the presence of HIV infection, where it is the most common cause of bacterial pneumonia [1]. Many of the behavioral and demographic factors that increase the risk for invasive pneumococcal disease (IPD) in the general population such as smoking, injection drug use (IDU), alcohol abuse, race, lung disease, and close contact with children are more common in HIV-infected populations [2]. However, the precise role and importance of these factors in the context of an HIV infected population has not been explored [2].

The incidence of IPD in HIV patients prior to the introduction of highly active antiretroviral therapy (HAART) was well described [3-6]. Shortly after the introduction of HAART in 1996 some, but not all HIV care centres, have reported a decline in the incidence of IPD in their patients [7-9]. During the early HAART era (between 1996 and 2003), incidence estimates of IPD in developed countries range from 379 to 820/100,000 person-years [6,9]; still occurring at a frequency of up to 35-100 times more than the HIV seronegative population [2,8]. The longer-term impact of HAART and of newer regimens on the incidence of IPD is not well established. In the general population, a decline in the burden of pneumococcal disease in adults has been reported after the introduction of the 7-valent pneumococcal conjugate vaccine (PCV7) for childhood immunizations due to "herd immunity" [10,11]. This may have also had a secondary and beneficial effect on incidence of pneumococcal infection in HIV patients [12].

As little is reported on the current incidence and morbidity of IPD in HIV-infected populations, we sought to examine the burden of pneumococcal disease in the new era of widespread use of HAART and active immunization programs in both HIV patients and the wider community.

## Methods

The Southern Alberta Clinic (SAC) and Calgary Laboratory Services (CLS) are the exclusive providers of HIV care and all routine laboratory services respectively in Calgary, Canada. Patients accessing care at SAC sign a voluntary informed consent form allowing administrative data to be used in research projects as approved by the University of Calgary Ethics Committee. Hospitalization data including discharge diagnoses from all city hospitals on SAC patients are collected and available within the SAC database [13]. Hospitalizations were included if a primary cause for admission was pneumococcal disease. Length of stay was evaluated using previously described methodology [13].

All HIV patients with cultures positive for *S. pneumoniae *growth between January 1^st^, 2000 and January 1^st^, 2010 were identified by reviewing the clinical database, which contains all of the microbiology results, provided to us by CLS. The results were reconciled for both completeness and for verification with hospitalization data. IPD was diagnosed when the culture was isolated from a normally sterile site such as blood, peritoneum, pleura, or cerebrospinal fluid (CSF); all such isolates were serotyped. Pneumococcal pneumonia was defined as isolation from a sputum sample or via bronchoalveolar lavage (BAL); most BAL isolates were also serotyped. Sputum and BAL samples were obtained because of strong clinical suspicion for lower respiratory tract infection, generally associated with radiologic evidence and severe illness.

The follow-up period was measured from the initial HIV-care visit until the patient moved, died, was disconnected from care, or until January 1^st^, 2010; this was used to calculate incidence rates [14]. If a patient presented with recurrent pneumococcal disease multiple times in one year, only the first instance was counted in an effort to reduce data distortion by a few patients with multiple recurrent cases. Clinical information obtained through routine care was evaluated with multivariate binary logistic regression. These risk factors included gender, age group (≤30, 31-45, 46-60, or ≥61), race (white, Aboriginal, black, or other), education (< high school or ≥ high school), a smoking history (if >1 month), alcohol abuse (>9 drinks/week for females, >14 drinks/week for males), IDU (at any time), nadir CD4 cell count (≤200, 201-500, or >500/μL), co-trimoxazole use, frequency of pneumococcal immunization (never, < every 5 years, or ≥ every 5 years), history of active tuberculosis (any clinical event), history of a common HIV-associated pulmonary disease (mycobacterium avium complex or *Pneumocystis Jiroveci *pneumonia), asthma, chronic obstructive pulmonary disease (COPD), or hepatitis C. All risk factors were adjusted for gender, age, a smoking history, IDU, and nadir CD4 cell count. These confounders were selected *a priori *based on expert opinion and previous studies [2,8,9]. Pearson's χ^2 ^and adjusted odds ratios with 95% confidence intervals (or P < 0.05) were used to evaluate significance. All demographic information was self-defined.

The effect of immunization with a 23-valent pneumococcal polysaccharide vaccine (PPV-23, Merck Frosst, Kirkland, Quebec, Canada) on the incidence of IPD was analyzed by comparing number of episodes during the total patient-time without immunization with the time post-immunization. The post immunization time was then sub-divided into categories: within three years of immunization, three to five years post-immunization, and greater than five years post-immunization. Any episodes of IPD within 30 days of immunization were to be excluded. Patients were immunized with two doses of PPV-23 five years apart and with a repeat dose every five years based on clinical discretion.

A nested case-control study was performed in patients who received PPV-23 comparing patients who developed IPD versus those who did not in a 1:3 ratio to evaluate the interaction of HIV-specific markers, while controlling for other known risks. Each patient diagnosed with IPD was randomly matched with three patients with the same age group, gender, smoking history, and history of IDU who also received PPV-23 but had not been diagnosed with IPD. Differences in HIV viral load and CD4 cell count at the time of immunization were evaluated with Levene's test for non-significance, followed by a 2-tailed t-test of means. An additional multivariate analysis included both CD4 count and viral load. Differences in nadir CD4 count, a history of COPD, asthma, and hepatitis C were also evaluated.

Statistical analyses were conducted with SPSS, version 15.0 for Windows (SPSS Inc., Chicago, Illinois).

## Results

Our data search revealed ten cases of pneumococcal disease (six IPD and four proven pneumococcal pneumonia) where HIV infection was diagnosed at the time of their presentation. These cases were excluded from further analysis, as they had not yet engaged in HIV care.

In a total of 1946 HIV patients in Southern Alberta over a ten-year period, there were 68 episodes of pneumococcal disease occurring in 50 patients. These included nine recurrent but distinct episodes of IPD (in six patients) that occurred within one year of a previous episode of IPD. There were 46 episodes of IPD; of these, 7 had positive sputum or BAL samples. There were 22 episodes of illness where *S. pneumoniae *was isolated from sputum or BAL only. Four patients died as a result of complications from IPD giving a case-fatality rate of 8.0% (95% CI: 2.4%-22%).

### Incidence

**Figure 1:**
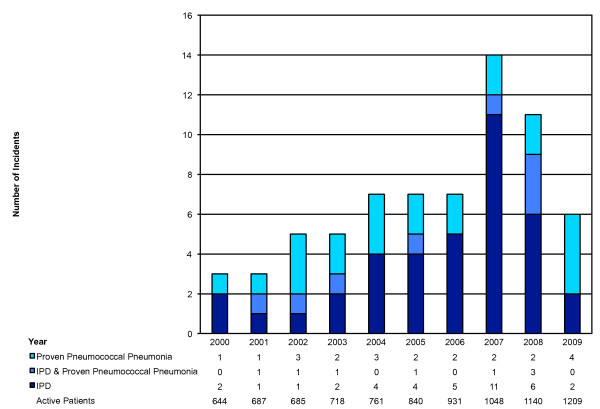
**Number and type of pneumococcal infections per year**. Pneumococcal disease in Southern Alberta HIV patients over 10 years; there is no evidence of a decreasing trend. The peak in 2007 overlaps with a serotype 5 outbreak among Calgary's homeless community [10]; number of active patients at the midpoint of each year.

There was a total of 11,099 person-years of clinic follow-up (median days/person: 2010, range: 3-3653); giving crude incidence rates of 342 and 613 cases per 100,000 person-years for IPD and all confirmed pneumococcal disease, respectively. A spike in 2007 was coincident with an outbreak of serotype 5 infections in the community (Figure 1) [10].

### Risks factors

Female gender (P < 0.01), age >60 (P < 0.0001), Aboriginal ethnicity (P < 0.05), lower education (P < 0.05), IDU (P < 0.01), smoking (P < 0.0001), nadir CD4<200/μL (P < 0.10), COPD (P < 0.05), and hepatitis C (P < 0.0001) were all independently associated with developing any pneumococcal disease in the multivariate analysis (table 1). Risk factors were similar for pneumococcal pneumonia and IPD. In ten patients with a splenectomy, there were no cases of IPD and one episode of non-bacteremic pneumococcal pneumonia.

**Table 1:** Risk factors for pneumococcal disease in HIV patients

**Risk Factor**		**IPD**		**Pneumococcal Pneumonia**		**All Pneumococcal Disease**	
	**n**	**n (%)**	**AOR (95% CI)**	**n (%)**	**AOR (95% CI)**	**n (%)**	**AOR (95% CI)**
				
**Gender**							
Female	418	10 (2.4)	2.2 (1.0-4.9)	10 (2.4)	3.4 (1.5-7.9)	16 (3.8)	2.6 (1.3-4.9)
Male	1528	22 (1.4)	1	18 (1.2)	1	34 (2.2)	1
**Age**							
≥61	58	3 (5.2)	9.7 (1.7-53.8)	4 (6.9)	45.4 (4.6-445.0)	6 (10.3)	22.5 (5.0-100.7)
46-60	476	11 (2.3)	2.5 (0.7-9.5)	4 (0.8)	2.9 (0.3-26.7)	12 (2.5)	2.8 (0.8-10.5)
31-45	1070	15 (1.4)	1.5 (0.4-5.5)	19 (1.8)	6.4 (0.8-49.2)	29 (2.7)	3.2 (0.9-10.8)
≤30	342	3 (0.9)	1	1 (0.3)	1	3 (0.9)	1
**Race**							
Aboriginal	233	13 (5.6)	3.0 (1.3-6.8)	7 (3.0)	1.5 (0.6-3.8)	16 (0.7)	2.1 (1.1-4.3)
Black	329	1 (0.3)	0.5 (0.1-3.9)	1 (0.3)	0.4 (0.0-3.7)	2 (0.6)	0.5 (0.1-2.3)
White	1225	18 (1.5)	1	19 (1.6)	1	31 (2.5)	1
Other	124	0	--	1 (0.8)	--	1 (0.8)	--
Unknown	35	0	--	0	--	0	--
**Education**							
<High School	437	15 (3.4)	1.8 (0.8-3.9)	15 (3.4)	2.2 (1.0-4.8)	24 (5.5)	1.9 (1.0-3.5)
≥High School	1155	15 (1.3)	1	13 (1.1)	1	24 (2.1)	1
Unknown	354	2 (0.6)	--	0	--	2 (0.6)	--
**IDU**							
Yes	231	13 (5.6)	3.3 (1.5-7.2)	7 (3.0)	1.4 (0.5-3.4)	17 (7.4)	2.5 (1.3-4.8)
No	1715	19 (1.1)	1	21 (1.2)	1	33 (1.9)	1
Smoker*							
Yes	1042	28 (2.7)	4.9 (1.6-14.7)	26 (2.4)	13.8 (3.1-60.9)	44 (4.2)	6.2 (2.5-15.3)
No	902	4 (0.4)	1	2 (0.2)	1	6 (0.7)	1
**Alcohol abuse†**							
Yes	266	7 (2.6)	1.4 (0.6-3.5)	7 (2.6)	1.6 (0.6-4.0)	11 (4.1)	1.4 (0.7-2.9)
No	1680	25 (1.5)	1	21 (1.3)	1	39 (2.3)	
**Nadir CD4/μL**							
≤200	875	27 (3.1)	5.1 (0.7-38.3)	22 (2.5)	1.5 (0.4-5.3)	41 (4.7)	2.8 (0.8-9.2)
201-500	836	4 (0.5)	1.0 (0.1-8.6)	3 (0.4)	0.2 (0.0-1.2)	6 (0.7)	0.5 (0.1-2.0)
>500	179	1 (0.6)	1	3 (1.7)	1	3 (1.7)	1
Not Available	56	0	--	0	--	0	--
**Any co-trimoxazole use**							
Yes	656	21 (3.2)	1.4 (0.6-3.4)	18 (2.7)	1.8 (0.7-4.8)	33 (5.0)	1.7 (0.8-3.5)
No	1290	11 (0.9)	1	10 (0.8)	1	17 (1.3)	1
**PPV-23 frequency**							
Never	429	9 (2.1)	1.7 (0.7-4.5)	2 (0.5)	0.3 (0.1-1.4)	10 (2.3)	1.0 (0.5-2.4)
< once per 5 years	472	12 (2.5)	2.3 (1.0-5.4)	12 (2.5)	1.8 (0.8-4.1)	20 (4.2)	2.1 (1.1-4.1)
≥ once per 5 years	1045	11 (1.1)	1	14 (1.3)	1	20 (1.9)	1
**History of pulmonary MAC or PCP**							
Yes	243	8 (3.3)	1.5 (0.6-3.6)	6 (2.5)	1.2 (0.5-3.2)	11 (4.5)	1.2 (0.6-2.6)
No	1703	24 (1.4)	1	22 (1.3)	1	39 (2.3)	1
**History of asthma**							
Yes	178	5 (2.8)	1.4 (0.5-4.0)	5 (2.8)	1.7 (0.6-4.8)	8 (4.5)	1.5 (0.7-3.5)
No	1768	27 (1.5)	1	23 (1.3)	1	42 (2.4)	1
**History of COPD**							
Yes	60	4 (6.7)	1.3 (0.4-4.3)	6 (10.0)	3.8 (1.3-11.0)	9 (15.0)	2.7 (1.1-6.4)
No	1886	28 (1.5)	1	22 (1.2)	1	41 (2.2)	1
**History of active TB**							
Yes	74	2 (2.7)	1.5 (0.3-6.8)	1 (1.4)	0.9 (0.1-7.0)	3 (4.1)	1.5 (0.4-5.3)
No	1872	30 (1.6)	1	27 (1.4)	1	47 (2.5)	1
**Hepatitis C**							
Infected	448	22 (4.9)	5.0 (2.0-12.1)	16 (3.6)	3.1 (1.3-7.7)	31 (6.9)	3.9 (1.9-7.9)
Not Infected	1498	10 (0.7)	1	12 (0.8)	1	19 (1.3)	1

* Self-reported smoking for greater than one month

† >9 drinks/week for females, >14 drinks/week for males

AOR, adjusted odds ratio; CI, Confidence Interval; IDU, injection drug use; PPV-23, pneumococcal polysaccharide vaccine; MAC, Mycobacterium avium complex; PCP, *Pneumocystis Jirovecii *pneumonia; COPD, chronic obstructive pulmonary disease; TB, tuberculosis

Variables are adjusted with logistic regression for gender, age group (≤30, 31-45, 46-60, or ≥61), a smoking history (if >1 month), IDU, and nadir CD4 cell count (≤200, 201-500, or >500/μL)

### Microbiology

**Figure 2:**
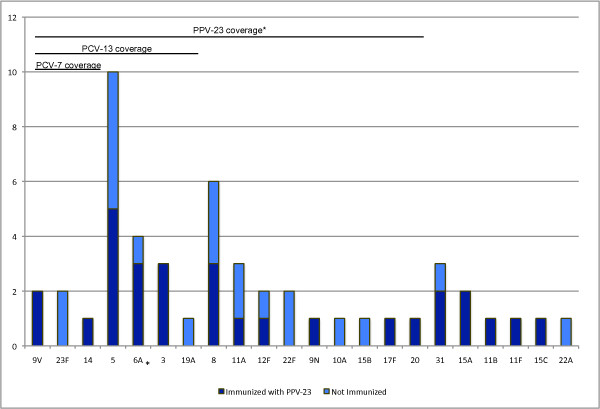
***S. pneumoniae *serotypes isolated over 10 years (n = 50)**. Isolated incidents of *S. pneumoniae *serotypes from 2000-2009 are described and arranged by coverage of three pneumococcal vaccinations. 40 were isolated from a sterile site only, 4 from bronchoalveolar lavage (BAL) only, and 6 from both a sterile site and BAL. Lines indicate serotypes included in the 7-valent pneumococcal conjugate vaccine (PCV7; n = 5), 13-valent pneumococcal conjugate vaccine (PCV13; n = 23), and the 23-valent pneumococcal polysaccharide vaccine (PPV-23; n = 37). *Serotype 6A is not included in PPV-23.

In 46 cases of IPD, *S*. *pneumoniae *was isolated exclusively in the blood in 43, once from ascitic fluid, once from both blood and ascitic fluid, and once from both blood and pleural fluid. *S. pneumoniae *was not isolated from the CSF or other sterile sites. Serotypes identified in sterile site cultures and BAL samples are shown in Figure 2. A specific *S. pneumoniae *serotype was identified in 55 of 60 total isolates (92%): 45 were from sterile sites, while 10 were isolated via BAL, and 6 were identified in a sterile site and via BAL on the same patient during the same incident. In one patient, two different serotypes (11A & 15B) were isolated from blood samples taken one day apart. Therefore, a total of 50 samples were included in the final analysis. PPV-23 covered 74% of all samples, including 19/29 (66%) of serotypes isolated from people who have previously been immunized and 18/21 (86%) of serotypes from people who had not been immunized (P = 0.10).

We also evaluated serotype trends over the course of the study period in the context of routine childhood immunization with PCV7, which was introduced in 2002 [15]. Before the introduction of PCV7 (January 1^st^, 2000 to December 31^st^, 2001), 1 of 4 incidents was due to a serotype covered by PCV7. In the immediate period the after the introduction of PCV7 (January 1^st^, 2002 to December 31^st^, 2004), 2 of 9 (22%) were PCV7 serotypes. After PCV7 was well established in our community (January 1^st^, 2005 to January 1^st^, 2010), 2 of 37 (5.4%) isolates were PCV7 serotypes; which represents a non-significant decrease compared to the period prior to 2005 (P = 0.10).

### Immunization

**Figure 3:**
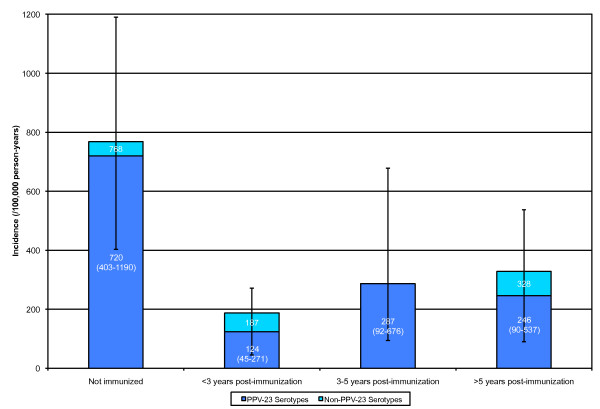
**Incidence of IPD with immunization**. Crude incidences with 95% Poisson confidence intervals are shown for PPV-23 serotypes. The incidence of IPD within three years of immunization was significantly lower than the incidence before immunization (P < 0.001). A composite incidence >3 years post-immunization incidence (not shown; mean: 262, 95% CI: 131-469) was also significantly lower than incidence before immunization (P < 0.01).

429 patients never received PPV-23 either because they declined immunization, were missed, or had CD4 counts below the recommended threshold. 1517 (78%) patients received PPV-23. Of these, 523 (34%) patients received two or more doses of PPV-23. Of a total 11,099 person-years of exposure for clinic patients, 2084 were prior to any PPV-23 immunization, and 9015 were post-immunization. 6578 person-years of follow-up were within 5 years after PPV-23 administration (4822 within 3 years) and 2437 follow up years were more than 5 years post-PPV-23 (Figure 3). There were no cases of pneumococcal disease within one month post-immunization. The incidence of IPD was significantly lower post-immunization for serotypes covered by PPV-23 (189 vs. 720 per 100,000 person-years, P < 0.0001) and for all serotypes (244 vs. 768 per 100,000 person-years, P < 0.001).

In a 1:3 case-control analysis of immunized patients, the case group (diagnosed with at least one incident of IPD, n = 19) had a significantly higher average log of viral load at the time of PPV-23 administration (P < 0.05). The mean (SD) of the log of viral load of cases and controls was 3.8 (1.2) and 3.1 (1.2), respectively. The viral load remained a significant predictor (P < 0.05) of IPD post-immunization when CD4 count at immunization was controlled for in multivariate analysis. CD4 counts were similar in the case group (mean: 430/μL SD: 190) and control groups (mean: 472/μL SD: 230, P = 0.44) at the time of immunization. No significant differences in nadir CD4, history of COPD, asthma, or hepatitis C were identified between groups (data not shown).

### Hospitalization

Hospitalization data were available to mid-2009 [13]. 45 of 64 (70%) cases of confirmed pneumococcal disease required in-patient care at the time of diagnosis, including 35 of 44 (80%) cases of IPD. The median length of stay when a primary diagnosis was pneumococcal disease was 7 days (range: 1-71 days).

## Discussion

The landscape of HIV care has changed dramatically over the last decade. The availability of newer and better-tolerated ART, the shift towards earlier therapy, and guidelines promoting more aggressive immunization in HIV infection have all contributed to improving morbidity and mortality. The effect of these improvements on IPD has not yet been scrutinized. Despite these advances in care, the incidence of IPD among our cohort has remained high.

In the United States, black race has been consistently correlated with increased risk of pneumococcal disease [9]. We identified that the highest rates of pneumococcal disease occurred in the Aboriginal population, but not in the black population, which locally consists mainly of recent migrants to Canada from Sub-Saharan Africa [16]. We are the first to report on the high incidence of pneumococcal disease in HIV-positive Aboriginal patients. Risk factors for pneumococcal disease identified are grossly otherwise consistent with previous studies [2,8,9]. While genetic factors may be important in this population in determining pneumococcal susceptibility or vaccine response, such differences have not been shown to be important in other ethnicities [17]. We suspect social and economic factors are more important and the pneumococcal epidemic in our population in 2007 may have particularly affected the disease incidence in this population.

Current WHO guidelines recommend immunization with PPV-23 for HIV patients in clinical stage 1 [18], while the CDC recommends immunization in those with a CD4 cell count greater than 200/μL [19]. Robust studies of PPV-23 in HIV-infected persons are lacking [2] and there has only been one randomized control trial of the PPV-23 effectiveness in HIV-infected patients, which showed harmful outcomes in a Ugandan population [20]. This study, however, did not report the use of ART and was undertaken at a time when potent HAART was unavailable to this population, making the trial results difficult to evaluate in the context of current care in the developed world.

The incidence of IPD with PPV-23 serotypes was 68% lower in those who had been immunized. By using the incidence of clinical disease, we found that the viral load at the time of PPV-23 immunization was the most significant predictor of PPV-23 failure to provide protection. This has been shown with other vaccines in HIV populations, in conjunction with CD4 cell count [18,21]. The effect of CD4 is more difficult to evaluate due to confounding use of antibiotic prophylaxis (usually including co-trimoxazole) against *Pneumocystis Jirovecii *pneumonia in patients with low CD4 counts.

Despite immunization, many of our patients developed IPD from serotypes included in PPV-23. This suboptimal protection in HIV patients has been previously recognized and led to a study investigating effectiveness of PCV7 in HIV-infected Malawian adults [2,22]. Caution however should be used in applying these results to a developed country with high rates of combination ART usage and childhood pneumococcal immunization programs. Furthermore, the placebo group had higher baseline viral loads, rates of tuberculosis and recurrent pneumonia, thus making the results difficult to interpret or extrapolate to developed world. We found that PCV7 would have covered only 10% of serotyped infections seen in our population over the last decade. A serotype shift after the implementation of a childhood immunization program is a plausible explanation for this low incidence as this has been described in HIV patients elsewhere, as well as the Calgary-area population [10,12]. Our results suggest that in adult HIV populations, there may be a trend away from serotypes included in the routine childhood vaccinations. Further shifts may occur after PCV13 was introduced into routine care in our region in 2010. Many of the serotypes observed in our study are included in PCV13, suggesting that a secondary benefit from reduction in pneumococcal disease among HIV-positive adults is possible.

Hospitalization is associated with a higher degree of severity and increased costs of care: proven pneumococcal disease usually required in-patient care. There is a high disease burden associated with pneumococcal disease in HIV-positive patients, causing long hospitalizations with high rates of morbidity and mortality despite widespread immunization with PPV-23, HAART, and free access to care.

This study has limitations as it was confined to a geographically defined population in Southern Alberta, Canada. Therefore, risk factors for IPD, circulating pneumococcal serotypes and their resistance patterns, and both clinic and community immunization practices as well as the general health of our HIV-infected population may differ from other populations or areas, however, risk factors were similar to other reports and geographically-defined populations also provide the most robust opportunity for understanding disease burden. We did not measure antibody levels to confirm response to PPV-23; however, infection rates are likely of greater clinical value. Furthermore, the analysis of immunization could not completely control for all social confounders associated with receiving the vaccine. Nevertheless, the time-since immunization approach, as well serotype analyses help control for these confounders.

## Conclusions

The burden of pneumococcal disease within HIV-infected patients remains high in the current era of HIV care, even in a population with universal access to both HAART and pneumococcal immunization. The ongoing burden of serious pneumococcal disease within HIV-infected persons reinforces the need for newer interventions and approaches such as reducing modifiable risk factors and enhanced multivalent conjugate vaccines to reduce pneumococcal-caused morbidity and mortality.

## Competing interests

JG has served on advisory boards for ViiVhealth, Gilead Abbott, Tibotec, Merck, and Bristol-Myers Squibb. RS and DG do not have any competing interests.

## Authors' contributions

RS designed the study, analyzed the data, and drafted and critically revised the manuscript. DG collected data and critically revised the manuscript. JG conceived the study, collected data, helped interpret findings, and critically revised the manuscript. All authors read and approved the final manuscript.

## Pre-publication history

The pre-publication history for this paper can be accessed here:

http://www.biomedcentral.com/1471-2334/11/314/prepub
